# Genome-wide comparative analysis of the *IQD *gene families in *Arabidopsis thaliana *and *Oryza sativa*

**DOI:** 10.1186/1471-2148-5-72

**Published:** 2005-12-20

**Authors:** Steffen Abel, Tatyana Savchenko, Maggie Levy

**Affiliations:** 1 Department of Plant Sciences, University of California, One Shields Avenue, Davis, CA 95616, USA

## Abstract

**Background:**

Calcium signaling plays a prominent role in plants for coordinating a wide range of developmental processes and responses to environmental cues. Stimulus-specific generation of intracellular calcium transients, decoding of calcium signatures, and transformation of the signal into cellular responses are integral modules of the transduction process. Several hundred proteins with functions in calcium signaling circuits have been identified, and the number of downstream targets of calcium sensors is expected to increase. We previously identified a novel, calmodulin-binding nuclear protein, IQD1, which stimulates glucosinolate accumulation and plant defense in *Arabidopsis thaliana*. Here, we present a comparative genome-wide analysis of a new class of putative calmodulin target proteins in Arabidopsis and rice.

**Results:**

We identified and analyzed 33 and 29 *IQD1*-like genes in *Arabidopsis thaliana *and *Oryza sativa*, respectively. The encoded IQD proteins contain a plant-specific domain of 67 conserved amino acid residues, referred to as the IQ67 domain, which is characterized by a unique and repetitive arrangement of three different calmodulin recruitment motifs, known as the IQ, 1-5-10, and 1-8-14 motifs. We demonstrated calmodulin binding for IQD20, the smallest IQD protein in Arabidopsis, which consists of a C-terminal IQ67 domain and a short N-terminal extension. A striking feature of IQD proteins is the high isoelectric point (~10.3) and frequency of serine residues (~11%). We compared the Arabidopsis and rice *IQD *gene families in terms of gene structure, chromosome location, predicted protein properties and motifs, phylogenetic relationships, and evolutionary history. The existence of an *IQD*-like gene in bryophytes suggests that IQD proteins are an ancient family of calmodulin-binding proteins and arose during the early evolution of land plants.

**Conclusion:**

Comparative phylogenetic analyses indicate that the major *IQD *gene lineages originated before the monocot-eudicot divergence. The extant *IQD *loci in Arabidopsis primarily resulted from segmental duplication and reflect preferential retention of paralogous genes, which is characteristic for proteins with regulatory functions. Interaction of IQD1 and IQD20 with calmodulin and the presence of predicted calmodulin binding sites in all IQD family members suggest that IQD proteins are a new class of calmodulin targets. The basic isoelectric point of IQD proteins and their frequently predicted nuclear localization suggest that IQD proteins link calcium signaling pathways to the regulation of gene expression. Our comparative genomics analysis of *IQD *genes and encoded proteins in two model plant species provides the first step towards the functional dissection of this emerging family of putative calmodulin targets.

## Background

The low solubility product constants of calcium phosphate salts provide a chemical rationale for the evolution of Ca^2+ ^as a universal second messenger. The necessity to decrease cytosolic Ca^2+ ^concentrations to submicromolar levels by exporting the cation into extracellular spaces or intracellular compartments that do not generate ATP, such as the endoplasmic reticulum or vacuole, creates a steep concentration gradient that allows for the controlled and gated generation of rapid Ca^2+ ^transients in response to extracellular stimuli. Such intracellular Ca^2+ ^signals are not only characterized by their magnitudes but also by their spatial and temporal resolution. The sum of these parameters is often referred to as the 'Ca^2+ ^signature' of a primary stimulus [1-4]. Numerous environmental cues of biotic and abiotic nature and endogenous physiological and developmental conditions trigger specific Ca^2+ ^signatures [2,5-8]. Stimulus-specific Ca^2+ ^oscillations are generated by voltage- and ligand-gated Ca^2+^-permeable channels (influx), and by Ca^2+^-ATPases and antiporters (efflux) to regain resting Ca^2+ ^levels [3,7]. Approximately 80 genes coding for potential Ca^2+ ^channels, pumps and antiporters have been identified in the Arabidopsis genome, suggesting complex generation and regulation of stimulus-specific Ca^2+ ^signatures [8].

Calcium spikes are recognized by several Ca^2+^-binding proteins and are decoded via Ca^2+^-dependent conformational changes in these sensor polypeptides and interacting target proteins [6,9-11]. Several classes of Ca^2+ ^sensors have been identified in plants that contain a Ca^2+^-binding helix-loop-helix fold known as the EF-hand motif. Calmodulin is the archetypal Ca^2+ ^sensor, which is exceptionally conserved in eukaryotes and contains four EF-hand motifs. About 250 EF-hand motif-containing proteins have been identified in Arabidopsis [12], including six typical calmodulins and 50 calmodulin-like proteins that differ significantly in sequence and number of EF-hand motifs [13,14]. Members of a second, plant-specific family of Ca^2+ ^sensors, which usually contain three EF-hand motifs, have similarity to the regulatory B-subunit of calcineurin in animals and are referred to as calcineurin B-like (CBL) proteins [9,15-17]. While calmodulins and CBL sensor proteins have no catalytic activity on their own and therefore are sometimes referred to as 'Ca^2+ ^sensor relays', a third major class of Ca^2+ ^sensors are bifunctional proteins, known as Ca^2+^-dependent protein kinases (CDPK), which contain a calmodulin-like domain with four EF-hand motifs and a Ca^2+^-dependent, Ser/Thr protein kinase domain on a single polypeptide chain [18,19]. Because of their dual functions as Ca^2+^-binding proteins and catalytic effectors the CDPK proteins are considered 'Ca^2+ ^sensor responders'. In Arabidopsis, CDPK and CBL proteins are encoded by multigene families of 34 and 10 members, respectively [16,19]. CDPKs play essential roles in hormone and stress signaling pathways as well as in plant responses to pathogens [20,21].

To transmit the information of the second messenger, Ca^2+ ^sensor relays such as calmodulins and CBL proteins interact with target proteins and regulate their biochemical activities. During the final phase of the transduction process, the target proteins modulate diverse cellular activities to establish the specific response to a given extracellular signal. The CBL sensor proteins interact specifically in a Ca^2+^-dependent fashion with a single family of SNF1-like Ser/Thr protein kinases, known as CBL-interacting protein kinases or CIPKs, which are encoded by 25 genes in Arabidopsis [16,22-24]. Current data indicate that CBL-CIPK interaction networks provide a signaling module for integrating plant responses to an array of environmental stimuli [17,23,25,26]. In contrast to CBL sensor proteins, which regulate a select set of target protein kinases, calmodulins interact with an astonishingly large number of target proteins. These have been extensively reviewed and include among other functional categories, proteins implicated in generating Ca^2+ ^signatures, enzymes in signaling and metabolic pathways, and transcriptional regulators [6,8,11,27-29]. The calmodulin-interacting domains of target proteins are not necessarily related in structure and exhibit high sequence variability, which may reflect the versatility of the calmodulin sensor relay. Nonetheless, calmodulin-interacting domains usually consist of a short (16–35 residues) basic amphiphilic helix, which is recognized by a flexible hydrophobic pocket that forms upon Ca^2+ ^binding to calmodulin [9,10,30,31]. Three calmodulin recruitment motifs are currently known although not all functionally characterized calmodulin-binding domains contain these specific motifs: the IQ motif (IQxxxRGxxxR; Pfam 00612) is thought to mediate calmodulin retention in a Ca^2+^-independent manner, whereas Ca^2+^-dependent interaction can be achieved by two related motifs, termed 1-5-10 and 1-8-14, which are distinguished by their spacing of bulky hydrophobic and basic amino acid residues [31-34]. Using various biochemical approaches, about 200 target proteins have been identified in Arabidopsis, a number that is expected to rise [8,11].

In a genetic screen for regulatory factors of the glucosinolate homeostasis in *Arabidopsis thaliana *[35], we have recently identified a gene coding for a calmodulin-binding protein with similarity to SF16 from sunflower [36]. We termed this protein IQD1 for the presence of a plant-specific domain of 67 conserved amino acids (referred to as IQ67 domain), which is characterized by a unique and repetitive arrangement of IQ, 1-5-10 and 1-8-14 calmodulin recruitment motifs. We demonstrated by biochemical and genetic studies that IQD1 is a nuclear calmodulin-binding protein that stimulates glucosinolate accumulation and plant defense [37]. In this study, we present a comparative genome-wide analysis of the entire *IQD *gene families in *Arabidopsis thaliana *(33 loci) and *Oryza sativa *(29 loci), which are predicted to encode proteins sharing the IQ67 domain. Our genomics analysis provides the framework for future studies to dissect the function of this emerging family of novel calmodulin target proteins.

## Results

### Identification and structure of *IQD *genes in *Arabidopsis thaliana*

**Figure 2:**
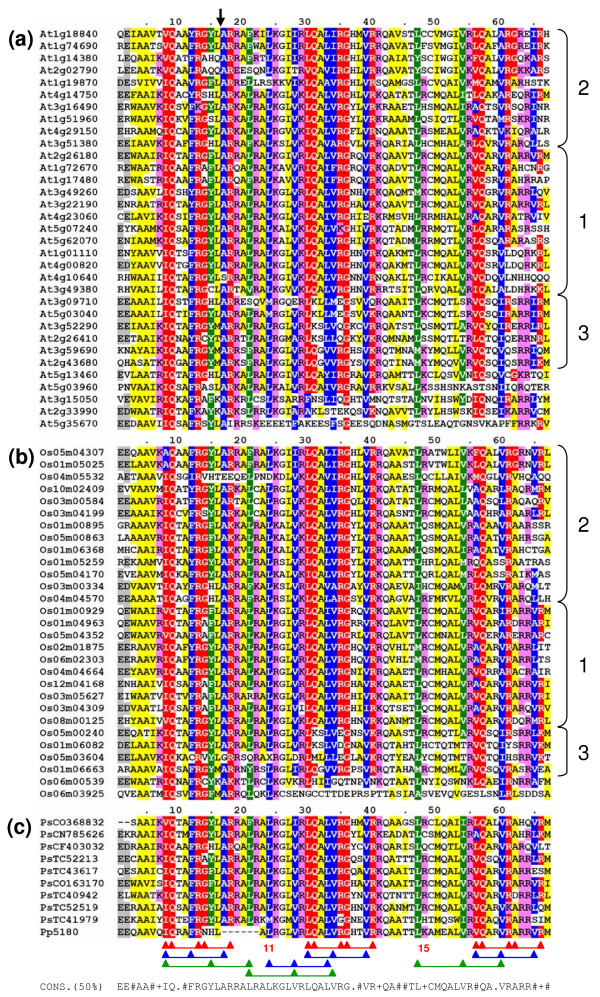
Amino acid sequence conservation of the IQ67 domain. Aligned are sequences of the IQ67 domain of 72 putative IQD proteins form *Arabidopsis thaliana ***(a)**, *Oryza sativa ***(b)**, *Pinus *spp. and *Physcomitrella patens ***(c)**. Each protein is identified by its gene identification (Arabidopsis and rice) or accession number (pine and moss). The numbers above the scheme (1–67) indicate the position within the domain as defined in this study. The position of the conserved phase-0 intron that separates the coding region of the IQ67 domain between codon 16 and 17 is marked by an arrow. The shading of the alignment presents residues (white text) of the IQ motifs (red), the 1-5-10 motifs (blue) and the 1-8-14 motifs (green). If a residue is part of more than one motif, the residue is shaded in the first assigned color as determined by the order of motifs listed above. In addition, acidic, basic and hydrophobic amino acid residues that are conserved in at least 50% of the 72 sequences are shaded in grey, pink and yellow, respectively. The scheme of connected triangles below panel C depicts the position and boundaries of the IQ (red), 1-5-10 (blue) and 1-8-14 (green) motifs. The consensus sequence at the bottom is based on the residues with greater than 50% conservation among the 72 proteins shown (#, hydrophobic; +, basic). Black braces at right indicate the major subfamilies as defined by the phylogenetic analysis of the 72 IQ67 domain sequences in Figure 7. Accession numbers of the putative pine and moss IQD proteins are given the prefixes 'Ps' and 'Pp', respectively.

In a previous study, we characterized IQD1 as a calcium-dependent calmodulin-binding protein and identified six closely related genes in Arabidopsis [37]. The encoded proteins share a conserved central region of 67 amino acid residues, referred to as the IQ67 domain, which is characterized by the occurrence of multiple calmodulin-binding motifs [32,33] that are arranged in a unique repetitive pattern. The IQ67 domain contains 1–3 copies each of the IQ motif (IQxxxRGxxxR or of its more relaxed version [ILV]QxxxRxxxx [R, K]), the 1-5-10 motif ([FILVW]x_3_[FILV]x_4_[FILVW]), and the 1-8-14 motif ([FILVW]x_6_[FAILVW]x_5_[FILVW]). In addition, several conserved basic and hydrophobic amino acid residues are flanking these motifs, and the IQ67 domain is predicted to fold into a basic amphiphilic helix ([37]; see Figure 2).

**Figure 1:**
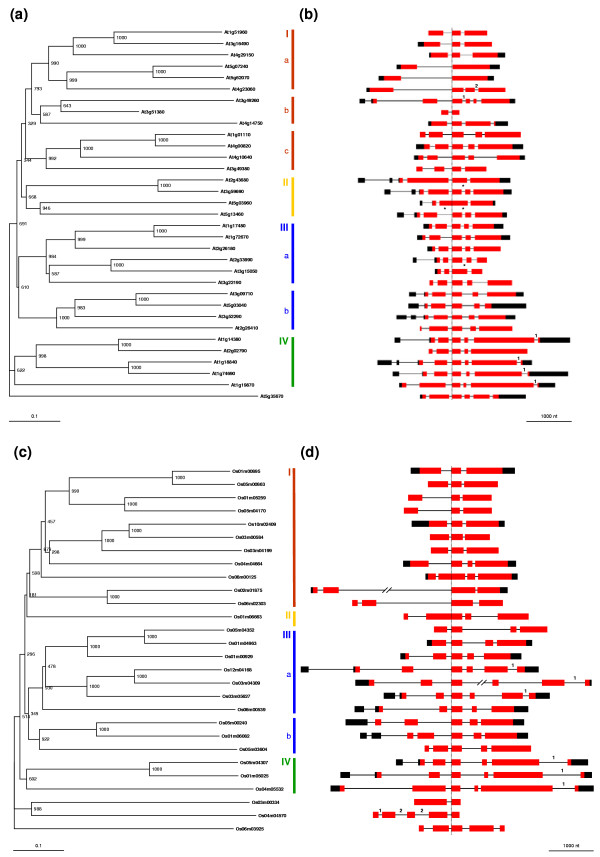
Phylogenetic analysis and exon-intron organization of *IQD *genes in *Arabidopsis thaliana *and *Oryza sativa*. Neighbor-joining trees of full-length amino acid sequences encoded by Arabidopsis **(a) **and rice **(c) ***IQD *genes are shown. The gene coding for the protein containing a C-terminally truncated IQ67 domain in Arabidopsis, At5g35670, and in rice, Osm0603925, was used as outgroup for each family. Bootstrap values (1,000 replicates) are placed at the nodes, and the scale bar corresponds to 0.1 estimated amino acid substitutions per site. Subfamilies and subgroups of *IQD *genes (I–IV) are highlighted by colored vertical bars on the right of the trees. The exon-intron organization of the corresponding *IQD *genes is shown for the Arabidopsis **(b) **and rice **(d) **gene family. Exons are depicted as boxes and introns as connecting thin lines. Protein-coding regions are colored in red, and non-translated regions, when supported by full-length cDNA sequences, are shown in black. The gene structures are drawn to scale and aligned along the left border (indicated by vertical dotted line) of the exon encoding amino acids 17–67 of the IQ67 domain, with the exception of At5g03960, Os08m00126 and Os01m06663 that have lost the respective intron. Additional intron losses are indicated by asterisks between Arabidopsis gene pairs. The exon-intron organization of the Arabidopsis *IQD *genes was taken from the TIGR Arabidopsis database, with the exception of At1g01110 for which the MIPS annotation was used as template. The presentation of the exon-intron organization of rice *IQD *genes was adapted to match the TIGR format of Arabidopsis *IQD *genes. The length of the second and third intron of Os02m01875 and Os03m04309 is 3.8 kb and 2.1 kb, respectively. Most introns of *IQD *genes are in phase-0. Six Arabidopsis and seven rice *IQD *genes contain phase-1 and phase-2 introns, which are labeled with the respective Arabic numeral. At2g02790, for which no full-length cDNA sequence is available, may also contain a phase-1 intron on its 3'end.

**Figure 5:**
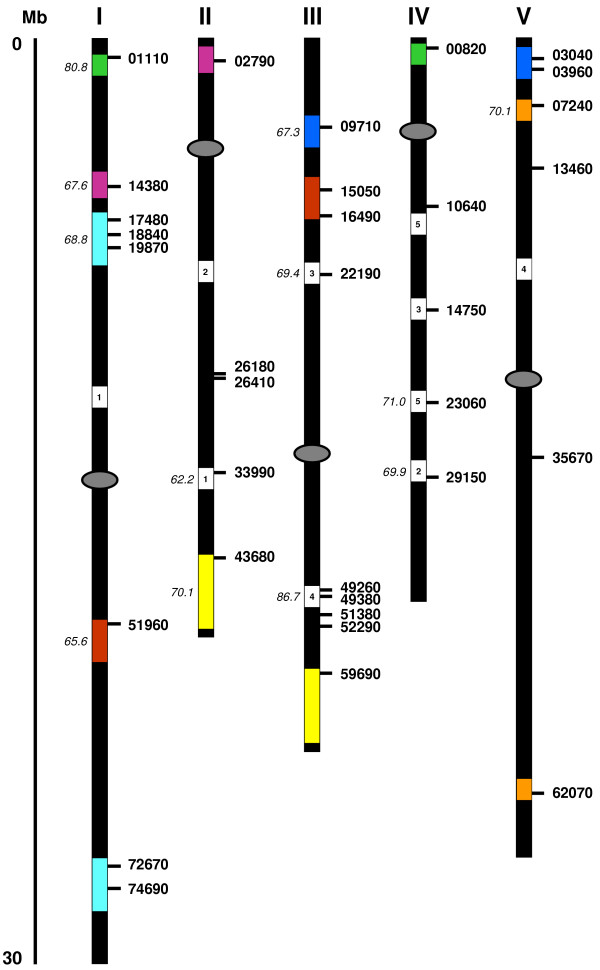
Chromosomal distribution and segmental duplication events for Arabidopsis *IQD *genes. The five chromosomes are indicated by Roman numerals and the centromeric regions by ellipses. Deduced chromosomal positions of the *IQD *genes are marked by horizontal bars and gene identification numbers (last five digits only). The scale is in megabases (Mb) and is adapted from the scale available on the TIGR database (see Materials and methods). Non-hidden duplicated chromosomal segments [48] that contain at least one retained *IQD *gene pair are color-coded. In three such segments (blue, brown, light blue), one sister *IQD *gene has been lost. Additional non-hidden duplicated segments that have lost sister *IQD *genes are shown in white and both segments are labeled with the same Arabic numeral. The duplicated segments of one such event (number 3) have likely experienced reciprocal *IQD *gene losses as the remaining genes, At3g22190 and At4g14750, are only distantly related (see Figure 1a). Numbers in italics at left indicate the estimated age (Myr) of the duplication event according to Simillion at al. [48]; the age estimates are given only once in the order of *IQD *gene location beginning with chromosome I.

To uncover the entire family of genes coding for IQD proteins in the Arabidopsis genome, we searched available Arabidopsis databases with multiple BLAST algorithms using full-length IQD1 (454 amino acids) and its IQ67 domain as the query sequences, followed by additional searches with related sequences (see Methods). In addition, we performed a pattern search with the IQ motif and its degenerate versions as the query sequences and inspected each hit for the presence of an IQ67 domain. We subsequently performed pair-wise sequence comparisons to exclude redundant entries from the initial data set, which is frequently caused by multiple identification numbers of the same DNA or protein sequence in the databases. A total of 33 non-redundant putative *IQD *genes were extracted from these sources (Table 1 and Figure 1). Full-length cDNA or EST sequences were available for 26 of those genes, and we attempted to clone by reverse transcriptase-mediated PCR cDNA sequences for the remaining seven genes. We succeeded to generate full-length cDNAs for three additional genes, At1g17480, At1g18840 and At4g23060, but were unable to amplify cDNAs for At1g51960, At2g02790, At3g22190 and At3g49380. To date, no evidence is available supporting the expression of At1g51960 and At3g49380 (Table 1). A comparison of the 29 genomic loci with their corresponding cDNA sequences revealed that most of the predicted gene models are correct, with only three exceptions (At4g10640, At2g26410, At1g01110). The full-length cDNA of At4g10640 encodes a protein that is 16 amino acid residues longer than the protein predicted by the MIPS MATDB annotation. This discrepancy is caused by the erroneous and superfluous annotation of a fifth intron in the last coding exon. For At2g26410, the translational start site and the 5' border of the first intron were misannotated for the MIPS MATDB entry when compared with its full-length cDNA. The available cDNA for At1g01110, annotated as a full-length cDNA (Arabidopsis TIGR db Annotation Version 5.0), encodes only three exons but is likely truncated at its 5'-end because (i) At1g01110 and At4g00820 are paralogous genes that evolved by a segmental duplication event (see Figure 1a and Figure 5), and (ii) the At4g00820 gene model of five coding exons is supported by a full-length cDNA sequence. We therefore consider the MIPS MATDB annotation of At1g01110 (five coding exons) to be correct. The gene models of At1g51960, At2g02790, At3g22190 and At3g49380 remain to be verified as no full-length cDNA sequences are available. Structural examination of the 33 putative *IQD *genes revealed the presence of 2–6 translated exons, suggesting that IQD proteins are quite diverse. Almost two-thirds of the gene family (20 members) contains more than four protein-coding exons, and 12 genes encode one or two non-translated exons in their 5'-region (Figure 1b). All introns of most *IQD *genes are phase-0 introns, separating exactly two triplet codons [38]. The last intron of At1g23060 is in phase-2, which lies between the second and third nucleotide of joining codons, and a phase-1 intron is found in five other *IQD *genes (Figure 1b). The average size of *IQD *genes in Arabidopsis is 2.4 kb (Table 3).

**Table 1:** The *IQD *gene family of *Arabidopsis thaliana*

**Gene Identifier**	**REFSEQ Accession**	**Protein ID**	**cDNA Accession^a ^Protein ID**	**Expression^b^**	**Protein Name^c^**	**Size (aa)**	**Mass (kD)**	**IP**	**Predicted_Location^d^**	
									**PSORT**	**TargetP**
At1g01110	NM_099993	NP_563618	AY085363*	A C D	IQD18	527	59.2	10.3	N	?
At1g14380	NM_101305	NP_563950	BT005935A AO64870	A B C D	IQD28	664	72.8	9.7	N	?
At1g17480	NM_101610	NP_173191	AY702665	A C D	IQD7	370	41.0	10.5	?	?
At1g18840	NM_101741	NP_173318	AY702666	A B C D	IQD30	572	62.7	9.2	N	?
At1g19870	NM_101842	NP_564097	BT001081A AN46862	A B C D	IQD32	794	86.8	5.2	N	C 0.65/4
At1g51960	NM_104077	NP_175608	-	-	IQD27	351	39.3	10.1	?	?
At1g72670	NM_105926	NP_177411	BT010652A AR07516	A C D	IQD8	414	45.9	10.3	N	?
At1g74690	NM_106127	NP_177607	AY128860A AM91260	A C D	IQD31	587	65.2	9.6	?	?
At2g02790	NM_126334	NP_178382	-	A C	IQD29	636	69.8	9.6	N	C 0.71/4
At2g26180	NM_128176	NP_180187	BX818988	C D	IQD6	416	46.9	10.5	N	?
At2g26410	NM_128198	NP_180209	BX840898	A	IQD4	527	58.3	10.3	?	?
At2g33990	NM_128950	NP_180946	AU237877 AV557487	A D	IQD9	249	28.5	10.8	N	?
At2g43680	NM_180068	NP_850399	BT008408A AP37767	A B	IQD14	668	74.3	11.3	?	?
At3g09710	NM_111805	NP_187582	AY827468	A C D	IQD1	454	50.5	10.4	N	?
At3g15050	NM_112367	NP_188123	BX825987	B C D	IQD10	259	29.6	10.3	?	C 0.91/1
At3g16490	NM_112520	NP_188270	BX824788	A D	IQD26	398	48.7	10.1	?	?
At3g22190	NM_113116	NP_188858	-	A	IQD5	400	44.5	10.1	N	?
At3g49260	NM_114785	NP_566917	BT000602A AN18171	A B D	IQD21	471	52.1	10.0	N	?
At3g49380	NM_114798	NP_190507	-	-	IQD15	352	40.8	10.2	N	?
At3g51380	NM_114997	NP_190706	BX838271 (FL-EST)	A D	IQD20	103	11.8	12.4	M	M 0.80/2
At3g52290	NM_115089	NP_190797	BT005639A AO64059	A B C D	IQD3	430	48.1	10.6	?	?
At3g59690	NM_115831	NP_191528	BT001176A AN65063	A D	IQD13	517	58.5	10.9	?	?
At4g00820	NM_116308	NP_567191	BX826435	A C D	IQD17	534	60.0	10.3	?	M 0.38/5
At4g10640	NM_117132	NP_192802	BT010145A AQ22614	A D	IQD16	423	48.7	10.1	N	?
At4g14750	NM_117560	NP_193211	BX827601	A C D	IQD19	387	43.9	9.7	?	?
At4g23060	NM_118435	NP_194037	AY702664	A B C D	IQD22	543	60.3	10.2	?	M 0.50/4
At4g29150	NM_119059	NP_194644	BT003896A AO41944	A D	IQD25	383	41.4	10.7	?	M 0.78/3
At5g03040	NM_120382	NP_568110	AY143972A AN28911	A B C D	IQD2	461	50.5	10.6	N	C 0.55/3
At5g03960	NM_120478	NP_196016	BX829656	-	IQD12	403	46.0	10.6	?	M 0.76/2
At5g07240	NM_120806	NP_196341	BT006056A AP04041	A C D	IQD24	401	45.3	10.3	?	M 0.54/4
At5g13460	NM_121349	NP_196850	AY128736A AM91136	C D	IQD11	443	50.8	10.0	N	?
At5g35670	NM_122958	NP_568529	AK128736B AD43467	C D	IQD33	442	49.5	8.5	?	M 0.47/5
At5g62070	NM_125600	NP_201013	AY143917A AN28856	A C D	IQD23	403	44.3	10.5	N	C 0.51/5

^a ^Full-length cDNAs (asterisk denotes a cDNA clone that is likely 5'-truncated).

^b ^Additional evidence for *IQD *gene expression provided by (A) whole-genome array [105], (B) community microarray data [94], (C) Massively Parallel Signature Sequencing (MPSS, [106]), (D) EST clones.

^c ^Nomenclature of *IQD *genes is arbitrary. Levy et al. [37] cloned *IQD1 *and reported closely related genes *IQD2-IQD6*. The designation of *IQD7-IQD33 *is based on the phylogenetic analysis presented in Figure 1a.

^d ^PSORT predictions: N (nucleus), C (chloroplast), M (mitochondrion). TargetP predictions: values indicate score (0.00 – 1.00) and reliability class (1–5; best class is 1).

**Table 3:** Average parameters of IQD genes and proteins from *A. thaliana *and *O. sativa*

	**Arabidopsis**	**Rice**
No. of genes	33	≥ 29
Gene length (kb)	2.4 ± 0.9	3.0 ± 1.6
No. of translated exons	4.5 ± 1.2	4.4 ± 1.2
Protein length (residues)	454 ± 132	471± 106
Molecular mass (kD)	50.8 ± 14.3	51.4 ± 11.8
Isoelectric point^a^	10.3 ± 0.6	10.4 ± 0.6
Frequency of Arg (%)^a^	9.3 ± 2.4	10.6 ± 2.5
Frequency of Lys (%)^a^	8.3 ± 2.3	5.9 ± 2.5
Frequency of Ser (%)	12.2 ± 2.2	10.2 ± 1.9
Frequency of Ala (%)	8.6 ± 2.2	12.8 ± 3.4

^a ^Computation does not include At1g19870 (pI of 5.2) and Os04m05532 (pI of 4.8).

### Predicted primary structure and properties of Arabidopsis IQD proteins

**Figure 3:**
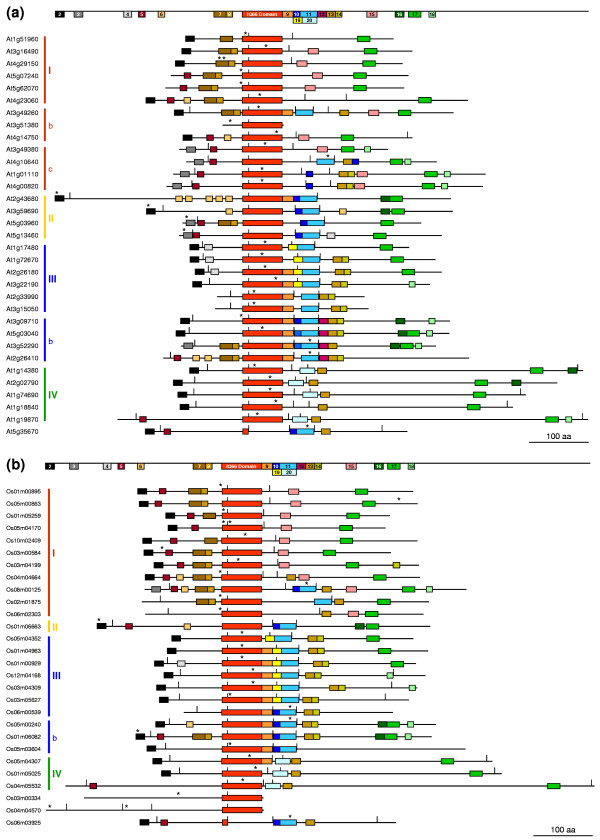
Motif patterns in IQD proteins of *Arabidopsis thaliana *and *Oryza sativa*. The schematic IQD proteins of Arabidopsis **(a) **and rice **(b) **are aligned relative to the IQ67 domain (orange box). Total amino acid sequence length, boundaries of protein-coding exons (vertical tick marks), and length and position of separate and distinct MEME motifs (shown as color-coded boxes) are drawn to scale. Motifs shared by the primary structures of at least four Arabidopsis IQD proteins are depicted at the reference bar on top of each alignment and numbered consecutively, beginning with motifs most N-terminal in the protein. Motif numbers are cross-indexed in Table 5 that lists the multilevel consensus sequence for each MEME motif. The position of putative calmodulin-binding sites predicted by the Calmodulin Target Database [40] (see Table 4) is indicated by an asterisk above each protein model. IQD proteins are aligned in the same order as they appear in the phylogenetic trees (see Figure 1). Subfamilies and subgroups (I–IV) of IQD proteins are highlighted by colored vertical bars next to the gene identifiers.

Having identified non-redundant and verified potential IQD protein coding sequences, we developed a set of criteria for the presence of the IQ67 domain in the 33 predicted Arabidopsis proteins. The IQ67 domain is characterized by the precise spacing of three copies of the 11-amino acid IQ motif, which are separated by short sequences of 11 and 15 amino acid residues (Figure 2a). The first IQ motif is best conserved (present in 32 proteins), followed by the second (26 proteins) and third (12 proteins) IQ repeat. Although the third IQ motif shows the highest degree of sequence degeneration, its initial hydrophobic amino acid and following glutamine residue are present in 31 proteins. Each IQ motif is congruent with a 1-5-10 motif of hydrophobic amino acids, which again is least conserved for the last IQ motif. A fourth 1-5-10 motif overlaps the first spacer sequence and second IQ motif. Each IQ motif also partially overlaps with a 1-8-14 motif. Besides these repetitive motifs, the IQ67 domain is characterized by the presence of additional conserved hydrophobic and basic amino acid residues flanking each IQ motif (Figure 2a). A hallmark of *IQD *genes is the presence of a phase-0 intron at an invariant position within the coding region of the IQ67 domain that disrupts codon 16 and 17 (equivalent to codon 9 and 10 of the first IQ motif). At5g03960 is the only exception to this rule, which encodes the entire IQ67 domain on its second and central exon (Figure 1b and Figure 3a). Given these criteria, 32 proteins contain at least two or three discernible IQ motifs with the accompanying 1-5-10 and 1-8-14 motifs in their IQ67 domain, which we therefore consider *bona fide *IQD proteins. The protein encoded by At5g35670 does not meet these criteria because it only contains the first, albeit truncated IQ motif provided by the N-terminal exon of the IQ67 domain (exon 2 of At5g35670). The exon coding for the remainder of the IQ67 domain (residues 17–67) is missing and replaced by an unrelated exon in At5g35670 (Figure 2a and Figure 3a). However, the At5g35670 protein shares five common amino acid sequence motifs outside the IQ67 domain with a large set of IQD proteins as detected by comparative MEME (Multiple Expectation Maximization for Motif Elicitation) analysis [39] of the complete amino acid sequences of the 33 Arabidopsis proteins (Figure 3a). As most of these motifs are unique to IQD proteins, we consider At5g35670 a member of the *IQD *gene family in Arabidopsis. Since amino acids 17–67 of the IQ67 domain are encoded by the second or third exon of *IQD *genes, the IQ67 domain contributes to the core region of most IQD proteins. An interesting exception is At3g51380, which is the smallest member of the IQD protein family in Arabidopsis and consists of a C-terminal IQ67 domain and a short N-terminal extension of 35 amino acid residues.

**Figure 4:**
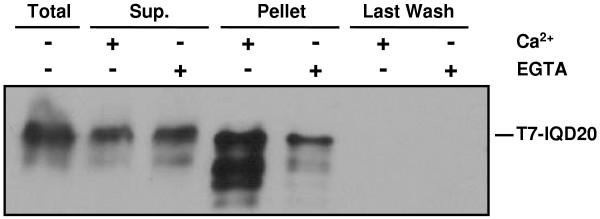
Interaction of Arabidopsis IQD20 and calmodulin *in vitro*. Calmodulin-agarose beads were incubated in the presence of Ca^2+ ^or absence of Ca^2+ ^(+EGTA) with soluble proteins prepared from induced bacterial cultures expressing a T7-tagged IQD20 protein and treated as described in Methods. Proteins of the total bacterial extract, the supernatant fraction, the entire pellet (beads) fraction, and of the last wash were resolved by SDS-PAGE, transferred to a membrane, and probed with a HRP conjugated T7-Tag monoclonal antibody.

Since At3g51380 is predicted to encode a 'minimal' IQD protein (IQD20), we tested whether calmodulin interacts with recombinant IQD20. We employed the same co-sedimentation assay that we recently used to demonstrate Ca^2+^-dependent binding of IQD1 to bovine calmodulin [37]. As shown in Figure 4, an epitope tagged T7-IQD20 fusion protein preferentially co-sedimented with calmodulin-agarose beads in the presence of Ca^2+^, whereas noticeably less T7-IQD20 protein was bound to immobilized calmodulin when the incubation mix and wash buffer were supplemented with EGTA. Thus, our data indicate that the smallest member of the IQD protein family in Arabidopsis interacts with calmodulin in a Ca^2+^-independent manner but suggest that calmodulin binding is possibly stimulated by the presence of Ca^2+ ^ions. We interrogated the web-based Calmodulin Target Database, which computes various structural and biophysical parameters of a given protein sequence to predict calmodulin binding sites [40]. This analysis predicted that IQD20 and all other IQD proteins of Arabidopsis contain, in addition to multiple IQ motifs, strings of high-scoring amino acid residues that indicate the location of putative calmodulin interaction sites (Table 4). The predicted calmodulin binding sites overlap with the IQ67 domain in 23 of the 33 IQD protein sequences (see Figure 3a).

**Table 4:** Predicted calmodulin-binding sites in Arabidopsis and rice IQD proteins

**Group^a^**	**Gene Identifier**	**Protein**	**Predicted calmodulin binding sequence^b^**
Ia	At1g51960	IQD27	(98) E**ERWAAVKIQKVFRG**SL (114)
	At3g16490	IQD26	(137) ALVRG**YLVRKRAA**ET (151)
	At4g29150	IQD25	(65) **KERRTHAIAVA **(75) (83) **DAAVAAAKAAAA **(94)
	At5g07240	IQD24	(105) E**YKAAMKIQSAFRGYL**A (121)
	At5g62070	IQD23	(115) QENI**AAMKIQSAFRGY**LAR (133)
	At4g23060	IQD22	(189) L**KGLVRLQAIVRGHIER**K (205)
Ib	At3g49260	IQD21	(137) RA**LRALK**GL (145)
	At3g51380	IQD20	(9) VV**RRKLLRRSQ**SR (22)
	At4g14750	IQD19	(155) A**LITLQAKAREQRIR**MIG (172)
Ic	At3g49380	IQD15	(140) ALVR**GHNVRRRTSITLQRVQAL**VRI (164)
	At4g10640	IQD16	(235) EI**AIKREKAQALALSN**QI (252)
	At1g01110	IQD18	(146) LV**KLQALVRGHNVR**KQ (161)
	At4g00820	IQD17	(157) LV**KLQALVRGHNVR**KQA (172)
II	At2g43680	IQD14	(1) **MVKKGSWFSAI **(11)
	At3g59690	IQD13	(1) **MGKKGSWFSAI **(11)
	At5g03960	IQD12	(8) FGW**MKRLFICEAKA**RAEK (24)
	At5g13460	IQD11	(5) **KGLFTVLKRIFISEVN **(20)
IIIa	At1g17480	IQD7	(125) IFR**GRQVRKQA**AVTLRC (141)
	At1g72670	IQD8	(119) VR**IQAIFRGRQ**VRK (132)
	At2g26180	IQD6	(116)VRG**RQVRKQAAVTLRCMQALVRVQAR**VRARR (146)
	At3g22190	IQD5	(137) QA**LVRVQARVRAR**RV (151)
	At2g33990	IQD9	(59) AYK**ARKSLRRLKGI**ARAKLS (78)
	At3g15050	IQD10	(61) RAFK**ARK**RLCS (71)
IIIb	At3g09710	IQD1	(103) GKSKEE**AAA**IL (113)
	At5g03040	IQD2	(141) VR**LKLLMEGSVVKQ**AAN (158)
	At3g52290	IQD3	(213) M**LNKQVATMRREKALAYA**F (231)
	At2g26410	IQD4	(245) RS**VNRKEASVRRERAL**AY (262)
IV	At1g14380	IQD28	(106) AHQ**ARRAF**RTL (116)
	At2g02790	IQD29	(159) VKV**QALVRGKKA**RSS (173)
	At1g74690	IQD31	(149) **LVRRQAVATLF **(160)
	At1g18840	IQD30	(159) G**IVRLQALARGREIRHSDIG **(178)
	At1g19870	IQD32	(230) ARR**ELLRSK**KVI (241)
	At5g35670	IQD33	(270) RER**ALAYA**FSQQL (282)
I	Os01m00895	OsIQD22	(134) PR**GRAAAVKIQTAFRGF**L (151)
	Os05m00863	OsIQD21	(434) N**RVQEAFNFKTAVVGRL**DR (453)
	Os01m05259	OsIQD20	(94) MVIQ**KAYRG**YLA (105)
	Os05m04170	OsIQD19	(87) **AVMIQKAFRGYLARRALRA **(107) (110) **LKALVKIQALVRGYLVRKQAATT **(129)
	Os10m02409	OsIQD18	(48) KK**RWSFRRS**SASASAAAM (65) (170) T**LRRMQALLVAQARlRA**Q (187)
	Os03m00584	OsIQD17	(28) **ALPGEAAKEKRWSFRRPVHG **(47)
	Os03m04199	OsIQD16	(138) K**LQALVRGHLVRRQA**S (153)
	Os04m04664	OsIQD15	(121) KRE**EYAAVRIQAA**FRG (136)
	Os08m00125	OsIQD14	(269) TR**KDAALKRERALS**YA (284)
	Os02m01875	OsIQD13	(127) ASRE**ERAA**VRIQ (138)
	Os06m02303	OsIQD12	(120) AGRE**ERAA**VRIQA (132)
II	Os01m06663	OsIQD11	(1) **MGKKGGWITA **(11)
**IIIa**	Os05m04352	OsIQD10	(114) RLV**RRQLAVTLKCMNA**LLR (132)
	Os01m04963	OsIQD9	(120) RGR**RVRKQL**AVTLKCMQALV (139)
	Os01m00929	OsIQD5	(143) QVRKQAAV**TLRCMQALVRVQARIRARR**VRMST (176)
	Os12m04168	OsIQD8	(147) AQA**RVRARRVR**ISL (160)
	Os03m04309	OsIQD3	(161) ARV**RARQVRVS**LE (173)
	Os03m05627	OsIQD4	(113) FLARR**ARR**ALKGL (125)
	Os06m00539	OsIQD7	(174) VKRE**RAMAYAFNHQWRA**R (191)
IIIb	Os05m00240	OsIQD1	(219) A**VRRERALAYAFSHQW**K (235)
	Os01m06082	OsIQD2	(1) **MGKKGNWFSAV **(11)
	Os05m03604	OsIQD6	(132) RVYLGRR**SQRARG**LDRL (148)
IV	Os05m04307	OsIQD23	(160) WL**IVKFQALVRGRN**VR (174)
	Os01m05025	OsIQD24	(155) LVRG**RNVRLS**GASI (168)
	Os04m05532	OsIQD25	(295) LV**RRQAA**ESLQ (305)
	Os03m00334	OsIQD26	(154) **GNAKLGRR **(161)
	Os04m04570	OsIQD27	(8) L**EKKRVITVQGRDKAGRP**I (26) (132) G**KLRYVSRLEYLWAHVRK**G (150)
	Os06m03925	OsIQD28	(252) **LAYAFSQQLRSCGGGGGGTT **(271)

^a ^Roman numerals correspond to subfamilies and subgroups of IQD proteins as used in Figure 1 and Figure 3.

^b ^Putative calmodulin-binding sites predicted by the Calmodulin Target Database [40] are shown for strings of amino acid residues with a score of at least "7". Residues with the highest score ("9") are highlighted in bold.

Although the predicted IQD proteins are quite diverse with respect to size (103–794 residues) and computed molecular mass (11.8–86.8 kD), they appear to be remarkably uniform in terms of their relatively high theoretical isoelectric point (10.3 ± 0.6), the only exception being At1g19870 (pI of 5.2), and with respect to the abundance of Ala (8.6 ± 2.2), Ser (12.2% ± 2.2%), and basic amino acid residues (Arg/Lys, 17.6% ± 2.2%). To uncover the possible subcellular localization of IQD proteins in Arabidopsis, we searched for different signature motifs specific to cellular compartments. Because of their high content of basic residues, and as suggested by PSORT, at least half of the IQD protein family (16 members) may be localized in the cell nucleus (Table 1). This conjecture is supported by the presence of several basic clusters in IQD proteins that conform to the SV40-type, MATα2-type, and bipartite type of nuclear localization signals [41], and by the nuclear localization of an IQD1-GFP fusion protein [37]. The remaining IQD proteins are predicted to be localized in the mitochondria (7), chloroplasts (5), or unknown compartments (Table 1).

### Chromosomal distribution and homology of Arabidopsis *IQD *genes

To infer clustering patterns that reflect IQD protein sequence similarity and evolutionary ancestry, we constructed phylogenetic trees by the neighbor-joining method [42] using IQD full-length sequences and the amino acid sequence of At5g35670 as outgroup. The At5g35670 gene encodes a C-terminally truncated IQ67 domain that lacks amino acid residues 17–67 (Figure 2a). The phylogenetic analysis of the Arabidopsis *IQD *gene family reveals four well-resolved subfamilies, two of which can be further divided into subgroups supported by the presence and position of introns, the occurrence of common protein motifs outside the IQ67 domain, and bootstrapping values (Figure 1a and 1b; Figure 3a). Large segmental duplications of chromosomal regions during evolution, followed by gene loss, small-scale duplications and local rearrangements, have created the present complexities of the Arabidopsis genome [43-51]. These events have likely shaped the size and structure of the current *IQD *gene family. We therefore analyzed the evolutionary history of *IQD *genes, which are relatively evenly distributed among all five Arabidopsis chromosomes (Figure 5 and Table 1). The topology of the phylogenetic tree (Figure 1a) suggests for several *IQD *genes in all subfamilies a clear paralogous pattern of gene divergence by gene duplication. Using the Arabidopsis Redundancy Viewer (MATDB), the Viewer of Segmental Genome Duplications (TIGR) and the searchable supplementary material provided by Blanc et al. [45] and Simillion et al. [48], we found that 26 of the 33 IQD genes are located in previously identified chromosomal duplications [45,47,48]. Eight pairs of duplicated *IQD *genes have been retained during evolution, whereas the *IQD *sister gene has been lost for each of the other 10 duplication events (Figure 5). All 18 duplications involving *IQD *genes occurred during the relatively recent genome-wide duplication event 75 ± 22 Myr ago, as estimated by Simillion et al. [48]. In most cases, the paralogous relationships indicated by segmental duplication are supported by the exon-intron organization and the phylogeny of the *IQD *gene pairs (Figure 1a and 1b). The following pairs of genes are therefore close paralogous *IQD *genes in Arabidopsis, sharing 50–67% amino acid sequence identity: At1g01110 and At4g00820; At1g14380 and At2g02790; At1g17480 and At1g72670; At1g18840 and At1g74690; At1g51960 and At3g16490; At2g43680 and At3g59690; At3g09710 and At5g03040; At5g07240 and At5g62070. Two orphan genes contained in opposite parts of a duplicated segment pair on chromosome III and IV, At3g22190 and At4g14750, group in different subfamilies of the phylogenetic tree and share substantially lower primary structure identity (20%) as well as less preservation of exon-intron organization (Figure 1a and 1b), suggesting reciprocal *IQD *sister gene loss after duplication of a chromosomal segment that contained two ancestral *IQD *genes. The genes At2g33990 and At3g15050 also appear to be closely related paralogs (Figure 1a, 43% identity); however they are positioned in different previously identified duplication segments, which points to a more complex evolutionary history. As expected, *IQD *genes of atypical structure (At5g03960, loss of intron in IQ67 coding region) or encoding atypical proteins (At1g19870, acidic pI; At3g51380, C-terminal IQ67 domain; At5g35670, truncated IQ67 domain) are either singleton genes (At5g35670, At3g51380), or orphan genes (At1g19870, At5g03960) whose homologous sister gene has been lost after duplication. Two pairs of closely positioned singleton genes, one each on chromosome III and IV, and two clustered genes in a duplicated segment on chromosome IV (At4g49260, At4g49380), suggest ancient tandem or local duplication events that have already resulted in substantial gene diversification (<30% identity for each gene pair). In summary, large-scale segmental duplication events appear to have exclusively contributed to the current complexity of the *IQD *gene family.

### Identification and predicted properties of the IQD protein complement in *Oryza sativa*

We next explored the occurrence and size of the *IQD *gene family in the extensively sequenced genome of rice [52,53]. BLAST searches in several databases of *O. sativa *ssp. *japonica *and *indica *(see Materials and methods) using several Arabidopsis full-length IQD protein sequences as the queries identified 29 different loci that encode non-redundant putative IQD proteins in rice. The general features of rice *IQD *genes and proteins are summarized in Table 2 and Table 3. Full-length cDNA sequences are available for 16 genes and generally support the respective gene model, with the exception of two loci (Os01m05259, Os03m04309) that are incorrectly annotated (see Table 2). The putative full-length cDNA sequences of two additional genes (Os01m06663, Os06m3925) are likely truncated in their coding region when compared with the conceptual translation products of each corresponding locus. A gene model could not be derived for the Os01m06368 locus in either *O. sativa *subspecies that covers the open reading frame of a corresponding partial cDNA sequence. To date, independent evidence for gene expression has been obtained for six of the remaining ten *IQD *family members for which a full-length cDNA is currently not available, suggesting that most *IQD *genes are functional in rice (Table 2). As for Arabidopsis, rice *IQD *genes encode 2–6 translated exons; however, less than half of the rice family members (13 genes) contain more than four exons (Figure 1d). Furthermore, all introns in most *OsIQD *genes are in phase-0; only six genes contain a phase-1 intron in their 3'-region and one gene (Os04m04570) is characterized by the presence of two phase-2 and one phase-1 intron in its 5'-region (Figure 1d). Rice *IQD *genes are slightly larger than Arabidopsis *IQD *genes, which is a result of increased intron length (Figure 1b and 1d; Table 3).

**Table 2:** The *IQD *gene family of *Oryza sativa*

**Gene Identifier^a^**	**Clone ID^b^**	**Position^c^**	**Protein ID Code^d^**	**cDNA Accession^e^**	**Expression^f^**	**Protein Name^g^**	**Size (aa)**	**Mass (kD)**	**IP**	**Predicted Location^h^**	
										**PSORT**	**TargetP**
Os01m00895	AP002743 02000445	70239–72382	NP_914546	AK119868	B	OsIQD22	465	49.7	10.5	N	M 0.69/3
Os01m00929	AP002746 02000453	152586–155207	NP_914588	AK073282	B	OsIQD5	442	48.9	10.3	?	?
Os01m04963	AP002901 02003727	7612–9986	NP_916574	AK102451	A	OsIQD9	441	48.2	11.0	?	M 0.55/5
Os01m05025	AP003288 02003743	38561–44222	9629.m05025^i^	AK062106	A B	OsIQD24	574	63.1	9.8	N	C 0.44/5
Os01m05259	AP003768 02003803	95943–99625	NP_916047^j^	-	A B	OsIQD20	378	42.4	10.7	?	?
Os01m06082	AP004366 02004199	106290–110032	BAD73780	AK072219	A B	OsIQD2	500	56.1	10.2	N	?
Os01m06368	AP003611 02004332	27187–28795	BAB63799^k^	AK120019*	-		n.d.	n.d.	n.d.	n.d.	n.d.
Os01m06663	AP003349 02004466	15479–17371	NP_915152	AK105622*	A	OsIQD11	563	61.7	11.5	?	?
Os02m01875	AP005534 02005830	59894–65564	XP_465098	AK105486	B	OsIQD13	485	52.0	10.4	?	?
Os03m00334	AC099399 02007792	57690–58691	XP_470188	-	B	OsIQD26	303	32.3	11.2	N	M 0.47/4
Os03m00584	AC105729 02029613	135566–136967	AAN06867	-	B	OsIQD17	417	44.3	10.2	?	M 0.61/4
Os03m04199	AC120505 02010452	144176–145684	XP_468989	-	-	OsIQD16	447	48.2	10.4	?	M 0.83/3
Os03m04309	AL731878 02014260	118442–126461	AK067192^l^	AAU89191	A B	OsIQD3	440	48.7	9.6	N	?
Os03m05627	AC084296 02011159	48853–52578	AAT75259	AK103438	A B	OsIQD4	422	47.0	9.8	?	?
Os04m04570	Chr.4^m^ 02014535	27592940–27594955	9632.m04570	-	-	OsIQD27	368	41.6	11.4	N	?
Os04m04664	AL607001 02017716	151253–153796	XP_473550	AK100392	A B	OsIQD15	464	50.1	10.4	N	M 0.42/5
Os04m05532	AL606999 02015015	85710–91604	XP_474230	AK066310	A B	OsIQD25	893	98.5	4.8	N	?
Os05m00240	AC093089 02015233	81361–85365	AAV33309	AK065809	A B	OsIQD1	474	52.0	10.2	?	?
Os05m00863	AC093954 02015642	45436–47015	XP_476075	-	-	OsIQD21	497	52.6	10.4	?	M 0.57/4
Os05m03604	AC108500 02017442	23568–26042	AAU90174	-	-	OsIQD6	538	57.8	9.6	N	?
Os05m04170	Chr.5^n^ 02017671	24971333–24973284	-	-	-	OsIQD19	367	40.5	10.8	N	?
Os05m04307	AC097112 02017716	54441–58756	XP_475770	AK101555	A B	OsIQD23	574	63.8	9.8	N	C 0.38/5
Os05m04352	AC104713 02017731	37570–40338	XP_475808	AK107193	B	OsIQD10	408	44.6	10.6	?	C 0.36/5
Os06m00539	AP004844 02018243	90441–94332	BAD69297	AK099462	A B	OsIQD7	353	39.4	10.4	N	?
Os06m02303	AP003572 02019223	18921–22322	BAD61625	-	-	OsIQD12	470	50.0	10.6	N	?
Os06m03925	AP0039440 2020217	329–2234	9634.m03925	AK109238*	-	OsIQD28	432	46.1	8.3	N	M 0.55/4
Os08m00125	AP005657 02022817	88345–90355	XP_479772	AK100461	A B	OsIQD14	543	59.0	11.0	?	?
Os10m02409	AC027662 02029613	17834–19903	NP_921513	AK110922	B	OsIQD18	485	52.2	10.3	N	M 0.73/3
Os12m04168	AL732532 02035326	133867–139274	9640.m04168	AK102525	A	OsIQD8	442	48.2	10.1	N	?

^a ^TIGR V2 pseudo-molecules annotation.

^b ^Upper line: nucleotide accession of a BAC clone coding for a rice *IQD *gene from *O. sativa *ssp. *japonica *[93]. Lower line: alternative rice BAC clone from *O. sativa *ssp. *indica *(the prefix, AAAA, is omitted).

^c ^Position of the *IQD *gene on the BAC clone from *O. sativa *ssp. *japonica*.

^d ^For four *IQD *genes, protein identification numbers are only available from the TIGR Rice Genome Project database [74].

^e ^cDNAs clones are full-length if not otherwise indicated. Asterisks denote cDNA sequences that are likely 5'-truncated by comparison with predicted mRNAs and encoded OsIQD proteins.

^f ^Additional evidence for expression provided by (A) EST clones and (B) Massively Parallel Signature Sequencing (MPSS, [106]).

^g ^Nomenclature of OsIQD genes is arbitrary. Levy et al. [37] cloned *AtIQD1 *and reported closely related rice genes *OsIQD1-OsIQD5*. The designation of *OsIQD6-OsIQD29 *is based on the phylogenetic analysis presented in Figure 1c.

^h ^PSORT predictions: N (nucleus), C (chloroplast), M (mitochondrion). TargetP predictions: values indicate score (0.00 – 1.00) and reliability class (1–5; best class is 1).

^i ^Region of Os01m05025 is not annotated on BAC clone AP003288 as indicated for AK062106 full-length cDNA sequence on KOME website [98]. Therefore, no Protein ID is available and the TIGR gene model accession is given instead.

^j ^Predicted gene model shows an N-terminal extension by 11 amino acids (possibly incorrect start codon), which was removed to meet consensus of IQD protein N-termini for computational analysis of protein properties.

^k ^Predicted protein of this gene locus is shorter for both *O. sativa *subspecies than the predicted polypeptide encoded by the partial cDNA clone that is truncated in the coding region N-terminal to the predicted IQ67 domain. Therefore, theoretical physico-chemical parameters of the predicted full-length protein could not be determined (n.d.).

^l ^Protein coding region is misannotated when compared with the predicted protein encoded by the full-length cDNA sequence.

^m ^BAC clone OJ1087C03 cannot be retrieved from GenBank.

^n ^Incorrect hyperlink from gene locus to BAC clone on RiceGE website.

Conceptual translation of full-length cDNA or predicted mRNA sequences and computation of theoretical physico-chemical protein parameters reveal that the IQD protein complement in rice is remarkably similar to the IQD protein family in Arabidopsis (Table 2 and Table 3). Comparative MEME analysis of the complete amino acid sequences of the 28 rice IQD proteins identified a similar set of conserved sequence motifs and their distribution along the polypeptide chain as found for members of the Arabidopsis IQD protein family (Figure 3b and Table 5). The IQ67 domain is positioned close to the core region of IQD polypeptides and is characterized by the same hallmarks as described for the Arabidopsis family, including the location and spacing of the three calmodulin-binding motifs (i.e., IQ, 1-5-10, 1-8-14), and the position of an invariant phase-0 intron that separates codon 16 and 17 of the IQ67 domain (Figure 2b and Figure 3b). As predicted by interrogation of the Calmodulin Target Database [40], all rice IQD proteins contain additional putative calmodulin binding sequences that often overlap with the IQ67 domain (Figure 3b and Table 4). It is interesting to note that the rice *IQD *gene family contains members with similar deviations from consensus properties as observed for the *IQD *gene family in Arabidopsis. These exceptions include loss of the phase-0 intron between the IQ67 domain-coding exons (Os01m06663, Os08m00125), replacement of the second exon coding for amino acids 17–67 of the IQ67 domain (Os06m03925), C-terminal location of the IQ67 domain (Os03m00334, Os04m04570), and an unusually large and acidic protein (Os04m05532). Since the rice IQD proteins display a similar range of structural and physico-chemical characteristics as the IQD family in Arabidopsis, it is very likely that we have identified most of the IQD family members in rice. Again, the majority of the family members (16 proteins) may be targeted to the cell nucleus; the remaining IQD proteins are predicted to be localized in the mitochondria (4), chloroplasts (1), or unknown compartments (Table 2).

**Table 5:** Major motifs in Arabidopsis and rice IQD proteins

**Motif^a^**	**Multilevel consensus sequence^b^**
1	EEWAAIKIQTAFRGYLARRALRALKGLVRLQALVRGHLVRKQAAMTLRCMQALVRVQAQVRR
2	MGKKGKWFKSLFGGF
3	SWFTAVKRIFISPTK
4	NKKWKLWRTSSED
5	EKRRWSFRKSS
6	PPCPPPPPPHH
7	KHAIAVAIATAAAAEAAVAAA
8	QAAAEVVRLTS
9	SEENQALQKQLHQKHHHE
10	GEDWDDSILSK
11	EEIEAKLQMRQEAAIKRERAMAYAFSHQW
12	WKNSSKTGNPTFMDP
13	DNPNWGWNWLERWMA
14	ARPWENRLMDD
15	YEENPKIVEMDTGKPYY
16	GSMNDDESFTSCPDF
17	PNYMANTESAKAKVRCQSAPR
18	SAKKRLSFPN
19	DHVKEIEEGWCDSIG
20	WMEKLTNNAFADKLLASSPTTLPLH

^a ^Numbers (1–20) correspond to the motifs schematically presented in the reference bars of Figure 3. Motif 1 corresponds to the IQ67 consensus sequence. The remaining motifs are listed in the order as they occur in the primary structures of IQD proteins, continuing with motifs most N-terminal.

^b ^Sequences were obtained from the MEME analysis of the 61 Arabidopsis and rice IQD full length proteins. Only consensus sequences that are shared by at least four Arabidopsis IQD proteins are listed.

### Chromosomal distribution of rice *IQD *genes

Unlike the Arabidopsis *IQD *gene family, which is evenly distributed over all Arabidopsis chromosomes, the distribution of *IQD *genes in the rice genome is clearly biased towards three chromosomes. Almost half of the rice *IQD *gene family members (14 loci) are contained in chromosomes I and V, and five genes are present on chromosome III. Three *IQD *genes are each found on chromosomes IV and VI, while seven of the twelve rice chromosomes contain either one or no *IQD *gene locus (Table 2). Such a heterogeneous distribution of *IQD *genes over the different rice chromosomes is consistent with an ancient aneuploidy event, which has been proposed to have occurred in rice about 70 Myr ago [51], and not with a whole-genome duplication or polyploidization event. Duplicated segments cover substantial regions of chromosome V (16%) and chromosome I (11%), the second and third largest fraction of segmental duplications after chromosome II (22%) [51]. The topology of the phylogenetic tree of *OsIQD *genes suggests four pairs of paralogous genes that evolved by segmental duplication (55–69% amino acid sequence identity); interestingly, three such pairs include *IQD *genes located on chromosome I and V (Figure 1c). Like the IQD protein family in Arabidopsis, the phylogenetic analysis of the rice gene family reveals four major subfamilies, and one can be divided into two subgroups. The two rice proteins containing the IQ67 domain at their C-terminus cluster as a separate subfamily (Figure 1c and 1d, Figure 3b).

### Comparative phylogenetic analyses

**Figure 6:**
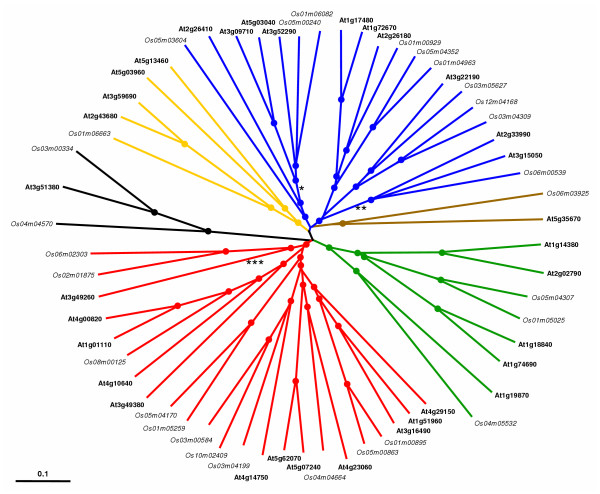
Phylogenetic relationships of *Arabidopsis thaliana *and *Oryza sativa *IQD proteins. The unrooted tree, constructed using ClustalX (1.81), summarizes the evolutionary relationship among the 61 members of both IQD protein families. The neighbor-joining tree was constructed using aligned full-length amino acid sequences. The scale bar corresponds to 0.1 estimated amino acid substitutions per site. Nodes supported by high bootstrap results (>75%) are indicated by dots. The same color code was used as in Figures 1 and 3 to highlight the different subfamilies (red, I; yellow, II; blue, III; green, IV; black, V [proteins with IQ67 domain on C-terminus]; brown, VI [proteins with truncated IQ67 domain]). The asterisks indicate the approximate position of branches corresponding to putative IQD proteins from pine (*TC522213, **TC41979, ***TC52519; Tentative Consensus of TIGR Unique Gene Indices).

We further investigated the relationship between the Arabidopsis and rice IQD protein families by generating an alignment of the 61 identified IQD amino acid sequences followed by the generation of a neighbor-joining phylogenetic tree (Figure 6). The combined phylogeny between the Arabidopsis and rice IQD sequences revealed six subfamilies of putative orthologous genes. Within each subfamily, the rice and Arabidopsis genes appear more closely related to each other than to *IQD *genes of the same species in a different subfamily, suggesting that an ancestral set of *IQD *genes already existed before the monocot-eudicot divergence. Four subfamilies of likely orthologous genes (I–IV) are composed of nearly identical sets of genes that constitute the respective subfamilies in Arabidopsis and rice (compare Figure 6 with Figure 1a and 1c). The remaining two subfamilies contain the genes encoding atypical IQD proteins in both species: At3g51380, Os03m00334 and Os04m04570 (IQ67 domain on protein C-terminus) are members of subfamily V, whereas At5g35670 and Os06m03925 (truncated IQ67 domain) comprise subfamily VI (Figure 6). The two genes coding for the acidic and unusually large IQD proteins, At1g19870 and Os04m05532 (Table 1 and Table 2), are members of subfamily IV and form a pair of orthologous genes. These subgroups of orthologous genes and other branches within the subfamilies are well-supported, which may be indicative for a relatively early diversification of *IQD *gene structure and function during plant evolution. The three genes that experienced loss of the conserved intron separating the IQ67 domain-encoding exons, At5g03960, Os01m06663 and Os08m00125, are members of different subfamilies (Figure 6), which suggests that intron loss occurred after the divergence of both evolutionary lineages. The phylogeny of Arabidopsis and rice *IQD *genes supports the occurrence of species-specific *IQD *gene duplications events. For example the two closely related *IQD *gene pairs in subfamily I (Os05m00863/Os01m00895 and At3g16490/At1g51960) or subfamily IV (Os05m04307/Os01m05025 and At1g18840/At1g74690) result from duplication events that occurred independently in both species.

To explore the evolutionary history of the *IQD *gene family in greater detail, we searched publicly available genomic and EST databases for homologous sequences in other plant species. We identified ESTs corresponding to IQD proteins for all angiosperm species represented in the TIGR Plant Gene Indices as well as for the gymnosperm *Pinus *ssp. (three putative full-length cDNA and six additional EST sequences). As expected, the putative full-length IQD proteins of pine (TIGR *Pinus *Gene Index entries TC41979, TC52213, and TC52519) are very similar to the Arabidopsis and rice IQD proteins with respect to calculated molecular masses (38.9–56.8 kD), isoelectric points (pI of 10.1–10.3) and frequencies of Ala, Ser, Arg, and Lys residues. A combined phylogenetic analysis of the Arabidopsis, rice and pine full-length IQD protein sequences reveals that the IQD proteins from *Pinus *cluster with different subfamilies (see Figure 6), suggesting that IQD proteins predated the evolution of vascular plants. We also performed a BLAST search of the moss database (see Materials and methods) and identified one contig EST sequence from *Physcomitrella patens *that encodes an IQD-like protein (contig5180). Although the deduced amino acid sequence appears to be truncated at the C-terminus (20 amino acid residues downstream of the IQ67 domain), an appreciable similarity with the protein encoded by At1g01110 is evident (33% identity), which includes the presence of MEME motif 3 at its N-terminus (data not shown). Interestingly, alignment of the deduced IQ67 domain of the moss polypeptide reveals a deletion of six residues that correspond to the N-terminus of the second IQ67 domain-encoding exon of most Arabidopsis and rice IQD proteins (Figure 2c). As the IQ67 intron is in phase-0 (see above) and since *A. thaliana *and *O. sativa *both express an *IQD*-like gene in which the second IQ67 domain-encoding exon is replaced by an unrelated exon, it is unlikely that the contig5180 DNA sequence is an artifact and probably represents either a novel variant of *IQD*-like genes or an ancestral gene of the *IQD *genes found in vascular plants.

**Figure 7:**
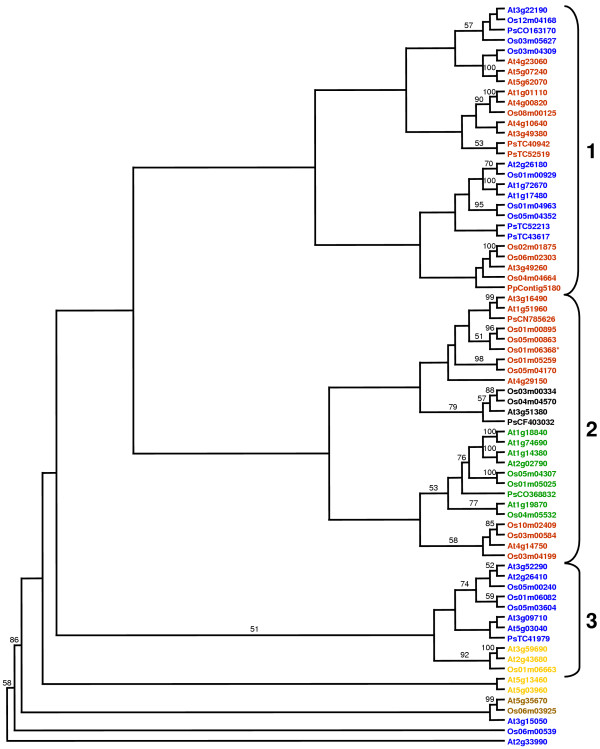
Phylogenetic relationships of the IQ67 domains encoded by *IQD *genes from *Arabidopsis thaliana*, *Oryza sativa*, *Pinus *ssp. and *Physcomitrella patens*. The unrooted tree was constructed from the alignment shown in Figure 2 using PAUP* 4.0 and the neighbor-joining method. Numbers on branches indicate the percentage of 1000 bootstrap replicates that support the adjacent node; low bootstrap support (<50%) was not reported. Black braces and Arabic numerals at right indicate the three major subfamilies as defined by the phylogenetic analysis of the 72 IQ67 domain sequences. Gene identification and accession numbers are colored using the same code as in Figure 6 to denote the different subfamilies of the parental IQD proteins. Accession numbers of the putative pine and moss IQD proteins are given the prefixes 'Ps' and 'Pp', respectively. The asterisk denotes the putative rice IQD protein for which a full-length amino acid sequence could not be predicted (see Table 2).

We finally examined the relationships between the IQ67 domains of the four plant species by constructing a neighbor-joining phylogenetic tree using the PAUP*4.0 program and the amino acid sequence alignment shown in Figure 2. Three major subfamilies of IQ67 domain sequences can be observed, which each contain members of the Arabidopsis, rice and pine IQD families. In addition, two small subfamilies and two single branches originate deeply in the unrooted tree and are only distantly related to the three major subfamilies, which can be further divided into subgroups (Figure 7). Bootstrap analyses indicated that the deep nodes of the tree have low statistical support, which may be attributed to the small size of the IQ67 domain. Low bootstrap support has also been observed for the phylogeny of the similarly sized DNA-binding domains of bHLH [54], Dof [55], or GATA [56] transcription factor families. Nevertheless, the IQ67 tree has better resolution in the outer clades. The short branches at the tips of the tree indicate high sequence conservation and strong evolutionary relationships among subfamily members. Interestingly, although the major subfamilies of IQ67 domain sequences (1–3) and of IQD full-length protein sequences (I–IV) overlap only partially (compare color code in Figure 6 and Figure 7), subgroups of IQ67 domain sequences largely correspond to subgroups of full-length IQD protein sequences as identified in Figure 6, which is suggestive of exon shuffling during the evolution of IQD proteins. We also investigated the effect of different programs and methods on IQ67 domain tree topology. Using ClustalX and the neighbor-joining algorithm or the PAUP*4.0 program and maximum parsimony analysis resulted in a similar tree topology (data not shown), which indicates that the neighbor-joining tree presented in Figure 7 is robust and reflective of likely phylogenetic relationships between IQ67 domains within subfamilies.

## Discussion

### The IQ67 domain – a plant-specific arrangement of putative calmodulin-interacting motifs

**Figure 8:**
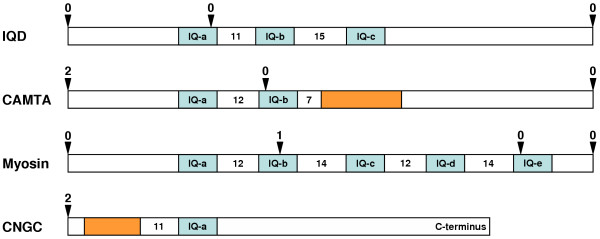
Organization of IQ motifs in major families of calmodulin-binding proteins. The scheme depicts the arrangement of the multiple IQ motifs present in proteins of the IQD family (this study; [37]), the CAMTA family of calmodulin-binding transcriptional activators [59-61], the myosin family [58], and the CNGC family of cyclic nucleotide gated channels [57, 104]. The IQ motifs are shown as light-blue boxes. Predicted and experimentally verified calmodulin-interacting peptide sequences are shown in orange. The numbers in the white spacers equal the number of separating amino acid residues. The triangles and numbers above each protein family model indicate the position and the phase of conserved introns, respectively. The positions of the left and right most introns are not drawn to scale.

In this study we characterized a possibly complete set of IQ67 domain-encoding genes in the current version of the *Arabidopsis thaliana *and *Oryza sativa *genomes. The defining features of the IQ67 domain are the invariant arrangement of three IQ motifs [32] separated by 11 and 15 intervening amino acid residues, and the conserved exon-intron organization (Figure 2). A pattern search of the Arabidopsis proteome with the conventional IQ motif (IQxxxRGxxxR) and its more generalized versions ([ILV]QxxxRxxxx[R,K]) as the queries confirmed a set of 33 *IQD *genes identified by reiterative BLAST searches. As expected from previous reports, our pattern search evidenced three additional major families and numerous miscellaneous proteins that contain at least one IQ motif: the CNGC family of cyclic nucleotide gated channels (20 members; [57]), the myosin family (17 members; [58]), and the CAMTA family of calmodulin-binding transcriptional activators (6 members; [59-61]). For each of these families, the spacing of IQ motifs and the exon-intron organization of the respective regions are unique and distinctive from the IQD family, which establishes the IQD proteins as a separate class of putative calmodulin targets of unknown biochemical functions (see Figure 8). The IQD proteins possibly constitute the largest class of putative calmodulin targets in plants. The size of the IQD family in Arabidopsis (33 proteins) and rice (29 proteins) clearly exceeds the size of other families of calmodulin-binding proteins [8] and is only comparable with the CIPK family (25–30 proteins) that interact with CBL Ca^2+ ^sensors in Arabidopsis and rice [16]. In addition to the IQ motif, the IQ67 domain contains multiple copies the 1-5-10 and 1-8-14 motifs, which are related and typified by their spacing of hydrophobic and basic amino acid residues. While the IQ motif is thought to mediate calmodulin retention in a Ca^2+^-independent manner, the 1-5-10 and 1-8-14 motifs are involved in Ca^2+^-dependent association of calmodulin with its target [33,34]. However, it should be noted that not all characterized calmodulin-binding domains contain these features [31,32].

We previously demonstrated that Arabidopsis IQD1 binds to bovine calmodulin in a Ca^2+^-dependent fashion [37]. In this study, we tested calmodulin binding for IQD20, the smallest member of the Arabidopsis IQD protein family (103 residues), which consists only of the IQ67 domain at its C-terminus and a short N-terminal extension of 35 amino acid residues. Interestingly, we observed interaction of recombinant IQD20 with calmodulin in the absence of Ca^2+^, which is possibly augmented when the metal ion is present (Figure 4). This observation and the prediction of putative calmodulin binding sites in IQD20 and all IQD proteins in Arabidopsis and rice, using the algorithm provided by the Calmodulin Target Database [40], strongly suggest that all IQD proteins have the potential to interact with calmodulin (Figure 3 and Table 4). Given our results with Arabidopsis IQD1 and IQD20, the prospect arises that different IQD proteins may interact with calmodulin in different modes, which could be Ca^2+^-independent, Ca^2+^-dependent, or more complex. The precise mechanism for each IQD protein is likely determined by the number and specific composition of the IQ, 1-5-10 and 1-8-14 motifs in the IQ67 domain, by the predicted calmodulin binding site adjacent to or overlapping with the IQ67 domain, and by the overall tertiary structure of the IQD protein. These structural features differ substantially between IQD1 and IQD20 (Figure 2, Table 1, Table 4), which are likely responsible for the observed differences in calmodulin interaction with respect to Ca^2+ ^dependency. The identification of interacting calmodulin or calmodulin-like proteins [14] and the biochemical characterization of calmodulin binding sites for each IQD protein are important tasks for future research.

It is interesting to note that the Calmodulin Target Database successfully predicts experimentally verified calmodulin-interacting peptides in CNGC [57] and CAMTA [59-61] proteins, which are located at conserved positions adjacent to the IQ motifs (see Figure 8). Although the IQ motif is likely as widely distributed as calmodulin and calmodulin-like proteins, the IQ67-specific arrangement of the three calmodulin retention motifs is confined to plant proteins and not found outside the plant kingdom, suggesting that this calmodulin-interaction module arose early in plant evolution.

### Evolution of IQD proteins

The presence of at least one putative *IQD*-like gene in *Physcomitrella patens *indicates that the IQD gene family originated during the early evolution of land plants, possibly before the divergence of bryophyte and vascular plant lineages 450–700 Myr ago [62], but not later than the split of gymnosperms and angiosperms about 300 Myr ago [63] as evidenced by EST and full-length cDNA sequences coding for at least nine *IQD *genes in pine. Molecular and phylogenetic analysis of *IQD *and *IQD*-like genes from ferns, bryophytes and green algae will be necessary to resolve the evolutionary origin of the *IQD *gene family.

To explore how the *IQD *gene family has evolved since the monocot-eudicot divergence 170–235 Myr ago [64], we performed a genome-wide comparative analysis of the *IQD *gene complement between Arabidopsis and rice. The phylogenetic trees of the 33 Arabidopsis and 28 rice *IQD *genes showed relatively long branches and closely clustered nodes, reflecting a high degree of sequence divergence, which is further indicated by the large variation in the number of protein-coding exons (2–6) and computed molecular masses of the predicted IQD proteins (Figure 1 and Tables 1, 2, 3). Based on their phylogenetic relationships, up to six different subfamilies of *IQD *genes can be defined for both species. This classification is supported by conserved exon-intron organization and protein motif patterns within each subfamily. The combined phylogenetic analysis revealed that members of all six subfamilies are present in the Arabidopsis and rice genome, indicating a relatively early diversification of the *IQD *gene family before the monocot-eudicot split (Figure 6). In those subfamilies, seven members of both *IQD *gene families are clearly recognizable as distinct orthologous pairs (e.g. genes coding for atypical IQD proteins), suggesting that the encoded proteins exert similar functions in both species. On the other hand, it is currently impossible to assign potential functions to *IQD *genes that are the result of recent species-specific duplication events leading to independent functional diversification.

The topology of the phylogenetic trees at the outer branches suggests that gene duplication played a prominent role in the evolution of both gene families, which is supported by the analysis of duplicated segments in the Arabidopsis genome (Figure 5). More than 80% of all genes in the annotated Arabidopsis genome reside in duplicated segments, and systematic analyses indicate that the Arabidopsis genome experienced a large-scale or even complete genome duplication event 30–90 Myr ago, sometime between the *Arabidopsis*-*Gossypium *and *Arabidopsis*-*Brassica *splits [48,49,51,65,66]. Evidence for older (>100 Mya) large scale-duplications exist, however, the frequency and precise timing of polyploidizations remains to be resolved and is a focus of current research [45,47-50,65,66]. The location of *IQD *genes in the Arabidopsis genome is clearly reflective of the recent large-scale duplication event. The *IQD *gene family is uniformly distributed among the five chromosomes, and 26 (or 79%) of the 33 *IQD *loci are found in duplicated segments of the recent age class (Figure 5). It is important to point out that 16 of those 26 genes in duplicated loci correspond to 8 *IQD *sister gene pairs, which represents an unusually high fraction of paralogous genes (44.5%) that have been retained from the extra gene set since the duplication event. Nonfunctionalization and subsequent gene loss is the most likely fate of a gene duplicate, and less than 27% of the entire paralogous gene set originating from polyploidy have been retained in Arabidopsis [45,48]. Preferential retention of duplicated genes has been observed for gene families in Arabidopsis with functions in signal transduction and transcriptional regulation [44]. Specific examples include the gene families encoding Aux/IAA (71.5% [67]), GATA (39% [56]) and GRAS (40% [68]) transcription factors, or genes coding for 20S proteasome subunits (64% [69]); the given percentages equal fractions of retained gene duplicates that we calculated from published data. Empirical evidence indicates that regulatory processes in metazoa such as signal transduction or gene transcription are dependent on gene dosage and stoichiometric protein-protein interactions [70]. As pointed out by Blanc and Wolfe [44], retention of a near-complete set or subset of duplicated genes coding for regulatory components such as transcription factors, kinases, phosphatases or Ca^2+^-binding proteins would minimize disturbances in sensitive stoichiometric and concentration-dependent relationships.

The evolutionary history of the rice genome is less understood. The view of an ancient polyploidy event has recently been questioned by evidence suggesting that rice experienced a partial or entire duplication of one chromosome about 70 Myr ago and can thus be considered an ancient aneuploid [43,51,52,71-73]. The observed non-uniform distribution of the 29-member *IQD *gene family in the rice genome, 50% of all *IQD *loci and three of the four paralogous *IQD *gene pairs are present on chromosomes I and V (Table 2), is more consistent with an aneuploidy than whole-genome duplication event. If polyploidization had occurred, it would be expected that *IQD *genes are randomly distributed over the whole rice genome, as observed for the *IQD *gene family in Arabidopsis. Given the significant differences in genome size and estimated gene count between rice (420 Mb, 57,900 genes [52,53,74]) and Arabidopsis (119 Mb, 27,500 genes [75]), the slightly larger size of the *IQD *gene family in Arabidopsis (33 members) versus rice (29 genes) is in agreement with a whole-genome duplication event in the evolutionary history of the Arabidopsis genome. A similar difference in membership has been reported for the Arabidopsis and rice gene families encoding Dof and GRAS transcription factors [55,68]. Nonetheless, *IQD *genes tend to be larger in rice than in Arabidopsis, which is mainly due to an increased intron length (Figure 1 and Table 3). In addition to polyploidization and segmental duplication events, tandem duplication is another important mechanism in the evolution of gene families [76] and plays a significant role in Arabidopsis as 17% of all genes are arranged in tandem arrays [48,77]. However, there is no evidence for tandem proliferation of the *IQD *gene families in the recent history of Arabidopsis and rice genomes.

Our analysis further suggests that exon shuffling played a major role during the evolution of *IQD *genes. Exon insertions and duplications, the major mechanisms of exon shuffling, contributed significantly to the complexities of eukaryotic proteomes [38,78,79]. A striking correlation between functional domains in protein and exons flanked by introns of matching phases, referred to as symmetrical exons, has been observed [38,80]. As stated by the phase-compatibility rules of exon shuffling [81], symmetrical exons and their flanking introns can be deleted, duplicated and inserted into introns of the same phase class without causing frame shifts. Thus, symmetrical exons flanked by introns of a single phase class tend to predominate in genes that largely evolved by exon shuffling and their nonrandom usage may be indicative of gene assembly by exon recruitment [38,78]. An intriguing feature of *IQD *gene organization in Arabidopsis and rice is the almost exclusive presence of symmetrical exons flanked by phase-0 introns (Figure 1). The strong bias for one intron phase class and the variation in the number of exons (2–6), and consequently size of the encoded proteins, is consistent with exon shuffling during the evolution of *IQD *genes. Exon shuffling is also suggested by the comparisons of patterns of protein motifs (Figure 3) and by the phylogenetic analysis of IQD full-length proteins and IQ67 domains, which indicate that phylogenetic relationships based on the IQ67 domain do not necessarily recapitulate patterns of protein and gene structure (Figures 5 and 6). Putative exon shuffling events may be recognized in some of the *IQD *gene structures. For example, At5g35670 and Os06m03925 encode a partial IQ67 domain and may have experienced exon swapping, or At4g10640 may have acquired its penultimate exon when compared with At3g49380 of the same subgroup (Figure 1). Exon shuffling may have played a prominent role in the diversification of *IQD *genes and their hitherto unknown functions. The above-mentioned gene families of transcription factors [55,56,67] contain introns of mixed phase classes, suggesting that exon shuffling played only a minor role during the evolution of these proteins with relatively defined functions. On the other hand, for example, all introns of genes coding for CIPKs are in phase-0 [16]. The exclusive usage of one phase class may indicate exon shuffling to generate the domain diversity necessary for kinase regulation and the ability to recognize a wide spectrum of protein substrates.

### Potential roles for IQD proteins

We have recently identified At3g09710 (*IQD1*) in a screen for Arabidopsis mutants with altered glucosinolate accumulation [37]. Glucosinolates are synthesized mainly by cruciferous species and constitute a class of secondary metabolites with roles in plant defense against pathogens and herbivores [35]. Characterization of gain- and loss-of-function alleles of *IQD1 *demonstrated that the encoded protein functions as a modulator of glucosinolate pathway-related gene expression. Tissue-specific expression of *IQD1 *is consistent with glucosinolate accumulation and mainly confined to the vascular tissues. We further demonstrated that an IQD1-GFP fusion protein is targeted to the cell nucleus and that recombinant IQD1 interacts with calmodulin in a Ca^2+^-dependent fashion [37]. It is therefore intriguing to hypothesize that IQD1 integrates intracellular Ca^2+ ^signals elicited by environmental cues such as herbivorous attack to fine-tune glucosinolate synthesis and accumulation. It should be pointed out that the rice genome does not contain an ortholog of At3g09710 (Figure 6), which is consistent with the absence of the glucosinolate pathway in this species and with functional diversification of the Arabidopsis and rice *IQD *gene families.

We are left to speculate on the biochemical and cellular functions of IQD proteins. One of the most intriguing features of IQD proteins is their high isoelectric point (~10.3), which has been maintained irrespective of protein size variation and domain composition, except for one family member each in Arabidopsis and rice. This observation suggests that the basic nature of IQD proteins is important for their biochemical functions. Although IQD proteins do not contain currently known DNA- or RNA-binding motifs, the basic isoelectric point and high frequency of serine residues, which are reminiscent of certain splicing factors [82], suggest that IQD proteins may associate with nucleic acids and regulate gene expression at the transcriptional or post-transcriptional level. Interestingly, we have recently observed that Arabidopsis IQD1 binds to nucleic acids (T. Savchenko, B. Zipp and S. Abel, unpublished results). A regulatory role for IQD proteins is also suggested by the relatively high fraction of retained duplicated *IQD *genes in the Arabidopsis genome. Preferential retention of paralogous gene pairs is thought to counteract disturbances in gene dosage and stoichiometric ratios of regulatory protein complexes after large-scale segmental duplication events and the onset of gene inactivation and loss of gene duplicates [44]. In this context, it is interesting to point out that the multiple Ca^2+^-dependent and Ca^2+^-independent calmodulin recruitment motifs of the IQ67 domains are likely involved in specific and cooperative interactions with calmodulins or calmodulin-like proteins. These interactions may dramatically alter the dynamic range of Ca^2+^-binding kinetics and, in turn, modulate interactions of the oligomeric protein complex with additional target proteins [31,83]. Many, if not most, members of the Arabidopsis and rice IQD protein families are likely to function in the cell nucleus (Tables 1 and 2). There is increasing evidence for the generation of nucleus-specific Ca^2+^-signatures in plant cells [1,84-86] and for a potential regulatory role of calmodulin and related Ca^2+ ^sensor proteins in nuclear processes such as transcription or gene silencing [9,60,61,87-90].

## Conclusion

We have systematically identified and characterized by bioinformatics a novel family of putative calmodulin target proteins in two model plant species, *Arabidopsis thaliana *and *Oryza sativa*. Our phylogenetic analyses indicate that the major *IQD *gene lineages originated before the monocot-eudicot divergence and that the expansion of the *IQD *gene family in the genomes of Arabidopsis and rice is consistent with a recent polyploidization and aneuploidization event, respectively. The extant *IQD *loci in Arabidopsis primarily resulted from segmental duplication and reflect preferential retention of paralogous genes, which is characteristic for proteins with regulatory functions. The almost exclusive usage of phase-0 introns and variable number of exons suggests a role for exon shuffling during the diversification of IQD proteins, which is also supported by phylogenetic relationships between the IQ67 domain and full-length IQD proteins. The unusually basic isoelectric point of IQD proteins and their frequently predicted nuclear localization suggest that IQD proteins link calcium signaling pathways to the regulation of gene expression. Our study provides a framework for the functional dissections of this emerging family of putative calmodulin target proteins.

## Methods

### Identification of *IQD *genes

To identify members of the *Arabidopsis thaliana *IQD protein family, multiple database searches were performed using the Basic Local Alignment Search Tool (BLAST [91,92]) algorithms BLASTP and TBLASTN available on the National Center of Biotechnology Information (NCBI) and The Arabidopsis Information Resource (TAIR) databases [93-95]. We used the amino acid sequence of IQD1 and of its IQ67 domain as initial query sequences, followed by the amino acid sequences of other IQD family members. Amino acid sequence pattern searches were performed on the TAIR website using Patmatch. Arabidopsis nucleotide and protein sequences as well as information regarding the gene structure were obtained from the Munich Information Center for Protein Sequences (MIPS) *Arabidopsis thaliana *Database (MATDB) [96], The Institute for Genomic Research (TIGR) *Arabidopsis thaliana *Database [74], and the *Arabidopsis thaliana *Plant Genome Database (AtPGD) [97]. To identify members of the rice (*Oryza sativa*) IQD protein family (OsIQD), we searched four different databases using the same BLAST algorithms. Sequences for *O. sativa *ssp.*japonica *were retrieved from the database at the TIGR Rice Genome Project [74]. Genomic sequences for ssp. *japonica *and ssp. *indica *were also obtained from the GenBank database containing the results of the International Rice Genome Sequencing Project and the draft rice genome sequence of the Chinese Academy of Sciences [53,93]. Rice full-length cDNA and EST sequences were searched in the Knowledge-based *Oryza *Molecular biological Encyclopedia (KOME) at the National Institute of Agrobiological Sciences [98] and in the TIGR Gene Indices [74]. Nucleotide and amino acid sequences as well as gene structure and chromosomal duplications were obtained from the same databases mentioned above. Genomic sequences that appeared to be misannotated by comparison with available cDNA sequences (full-length cDNAs, ESTs) were corrected for subsequent analysis. Sequences encoding putative IQD proteins in *Pinus *ssp. and *Physcomitrella patens *were identified by BLAST searches of the TIGR Gene Indices [74] and of the moss database NIBB PHYSCObase [99].

### Chromosomal duplication in the Arabidopsis genome

For the detection of large segmental duplications, we used the redundancy viewer at the MATDB [96], the duplicated blocks map provided by TIGR [74], the interactive supplementary material by Simillion et al. [48], and the interactive maps of duplicated blocks in Arabidopsis by Blanc et al. [45].

### Computational analysis of IQD proteins

The amino acid sequences of all IQD proteins were analyzed for physico-chemical parameters (ProtParam) and predicted subcellular localization (PSORT, TargetP) on the ExPASy Proteomics Server [100]. MEME (Multiple Expectation Maximization for Motif Elicitation) was used to identify conserved motif structures among IQD protein sequences [39]. Putative calmodulin-binding sites in IQD protein sequences were predicted by the Calmodulin Target Database [40].

### Alignment and phylogenetic analysis of IQD sequences

Multiple alignments of amino acid sequences were performed using ClustalW [101] or ClustalX [102] and were manually corrected. For generating the phylogenetic trees of full-length IQD protein sequences reported in Figures 1, 2 and 5, we used ClustalX (1.81) and the neighbor-joining algorithm [42]. Bootstrap analysis with 1,000 replicates was used to evaluate the significance of the nodes. The trees of the Arabidopsis and rice IQD protein families were rooted using each atypical protein containing a truncated IQ67 domain as an outgroup; an unrooted tree is shown for the combined analysis of all Arabidopsis and rice IQD proteins (Figure 6). For the creation of the unrooted phylogenetic tree of IQ67 domain sequences in Figure 7, we used in addition the PAUP*4.0 (b10) program to perform distance and parsimony analyses [103]. The same program was used for subsequent bootstrap analysis with 1,000 replicates to evaluate tree topology.

### cDNA cloning

The identification and cloning of a full-length cDNA for At3g09710 has been described previously [37]. Using similar conditions for reverse transcriptase-mediated PCR, we amplified predicted full-length cDNA sequences for

At1g17480 (forward: 5'-ATGGGTGGGTCAGGAAATTGGATT-3';

reverse: 5'-TTAGCTTCGCTGGCTCTTGG-3'),

At1g18840 (forward: 5'-ATGGGAAAGCCTGCAAGGTG-3';

reverse: 5'-TAACCGTTTCCTTCTCGGGACGA-3'), and

At4g23060 (forward: 5'-ATGGGAAAAGCGTCCCGGTGGTT-3';

reverse: 5'-TCAGTACCTATACCCAATTGGCATCC-3').

The resulting PCR products were subcloned into the vector pGEMT (Promega, Madison, WI) by TA cloning followed by DNA sequencing of the insert with T7 and SP6 primers.

### Expression of AtIQD20 and calmodulin binding assay

A full-length cDNA fragment encoding the predicted IQD20 protein of Arabidopsis was generated by RT-PCR using gene-specific primers

At3g51380 (forward: 5'-CGCGGATCCATGGCCAACTCCAAACGTTTG-3') and At3g51380 (reverse: 5'-GAGGAATTCTTAATGAGAGAG-3'). The PCR fragment was subcloned into the *Bam*HI and *Eco*RI sites of vector pET21a (Novagen, Madison, WI, USA), which provides an N-terminal T7-epitope tag. Expression of recombinant T7-IQD20 and calmodulin-binding assays using calmodulin-agarose beads (phosphodiesterase-3':5'-cyclic nucleotide activator from bovine brain; Sigma-Aldrich, St. Louis, MO, USA) were performed as previously described [37].

## Authors' contributions

SA carried out most of the bioinformatics analyses and wrote the entire manuscript. TS demonstrated calmodulin binding of IQD20. TS and ML contributed to data collection and IQD sequence analysis.
